# Therapy formulation for preventing progression of reversible to irreversible (FRANK) Alzheimer’s disease (FAD): A neurodegenerative disease

**DOI:** 10.1002/alz70859_099189

**Published:** 2025-12-25

**Authors:** Vincent O. Olughor, Kemi O Charles‐Okhe, Amany K Ladagu

**Affiliations:** ^1^ Faculty of Pharmacy, University of Ibadan, Ibadan Nigeria; ^2^ University of Ibadan, Ibadan Nigeria

## Abstract

**Background:**

The onset of FAD is marked by acute‐elevation of plasma‐24SHydroxylcholesterol. The risks factors driving progression of subclinical‐ to frank‐ Alzheimer’s disease (FAD) are: the presence of persistent food‐based micronutrients deficiencies, sub‐normal endogenous anti‐oxidants levels in plasma, presence of persistent exposures to lipophilic‐neurotoxins and the presence of evidence of dysregulation of autophagy. These risks factors cause a cocktail of many physiological disruptions and when the disruptions include Wallerian‐type degeneration of cholinergic neurones axons in the brain it leads to the development of frank Alzheimer’s disease (FAD) in the long run. Hence a therapy formulation for preventing these risks factors from causing Wallerian‐type degeneration of cholinergic neurons axons is a significant option for preventing FAD.

**Method:**

Through threaded literature reviews of the significance of the risk factors and the intervention therapy for reverting them to normal and maintaining their normal levels as the best option for preventing frank AD, caused by depletion of the neurotransmitter, acetylcholine and whose onset begins with memory loss.

**Result:**

Micronutrients deficiencies (MDs) of calcium/iron/vitamin‐D forces absorption of neurotoxic‐lead that will eventually cross the blood‐brain barrier (BBB). Neurotoxins absorbed following other MDs are also known. Sub‐normal endogenous anti‐oxidants levels in plasma cause certain lipophilic‐neurotoxins to disrupt the integrity of the BBB so that the same/other lipophilic‐neurotoxins can breach the BBB reach the brain causing multivariate‐disruptions including Wallerian‐type degeneration of cholinergic neurones axons in the brain. Autophagy, a vital cell process, plays a role in nutrients sensing and metabolisms, highlighting its significance in mediating the effects of dietary interventions on cellular functions and overall health. Dysregulation of autophagy contributes to such conditions as Alzheimer’s, Parkinson’s, Huntington’s diseases, diabetes, cancer and more.

**Conclusion:**

A Nutraceutical therapy containing full‐compliments of all essential (macro/micro/phyto)‐nutrients and dedicated to impact pH 7.35‐7.45 to plasma will be designed/formulated to prevent FAD from happening.

